# The Potential of *Agaricus bisporus* in Mitigating Pesticide-Induced Oxidative Stress in Honey Bees Infected with *Nosema ceranae*

**DOI:** 10.3390/life14111498

**Published:** 2024-11-17

**Authors:** Stefan Jelisić, Zoran Stanimirović, Marko Ristanić, Đura Nakarada, Miloš Mojović, Dušan Bošnjaković, Uroš Glavinić

**Affiliations:** 1 Department of Biology, Faculty of Veterinary Medicine, University of Belgrade, Bul. oslobodjenja 18, 11000 Belgrade, Serbia; 2 EPR Laboratory, Faculty of Physical Chemistry, University of Belgrade, Studentski trg 12-16, 11158 Belgrade, Serbia; djura@ffh.bg.ac.rs (Đ.N.); milos@ffh.bg.ac.rs (M.M.); 3 Department of Physiology and Biochemistry, Faculty of Veterinary Medicine, University of Belgrade, Bul. oslobodjenja 18, 11000 Belgrade, Serbia

**Keywords:** honey bees, oxidative stress, *Nosema ceranae*, pesticides, *Agaricus bisporus*, deltamethrin, EPR

## Abstract

Global climate change, environmental pollution, and frequent pesticide use severely reduce bee populations, greatly challenging beekeeping. Pesticides such as deltamethrin, a pyrethroid insecticide commonly used to control mosquitoes, can kill individual bees and entire colonies, depending on the exposure. Due to mosquito resistance to pyrethroid insecticides, components that enhance their effect are commonly used. This study explores the potential of *Agaricus bisporus* mushroom extract in mitigating oxidative stress in bees triggered by pesticides and *Nosema ceranae* infection. Our findings indicate that *A. bisporus* extract significantly reduced mortality rates of bees and spore counts of *N. ceranae*. Furthermore, the extract demonstrated antioxidant properties that lower enzyme activity related to oxidative stress (CAT, SOD, and GST) and MDA concentration, which is linked to lipid peroxidation. These results indicate that natural extracts like *A. bisporus* can aid bee health by mitigating the effects of pesticides and pathogens on honey bees, thus improving biodiversity.

## 1. Introduction

The European honey bee (*Apis mellifera*) is one of the most important pollinators of plants, directly affecting all agricultural production and biodiversity [1]. In addition to their role as essential pollinators, honey bees are valued for products such as honey, wax, and propolis, which have broad applications in food, medicine, and industry. In recent decades, agriculture has reduced pollen diversity, which has affected bee nutrition as a significant stressor [2,3].

Bee colony loss is a big problem of modern beekeeping, long occupying the attention of beekeepers and researchers. Global climate changes, environmental pollution, and the frequent use of pesticides, especially agricultural, are the most important disruptors of the ecosystem, crop production, and, therefore, the production of high-quality bee food [2,4,5]. Global changes in the dynamics of flowering, the quantity and quality of pollen and nectar, and the practice of growing monocultures (e.g., corn), which are of poor quality for bees, further increase the risk of protein starvation of bees [2]. *Nosema apis* and *N. ceranae* are common bee parasites. *Nosema apis* parasitise the European honey bee (*A. mellifera*), and *N. ceranae* parasitizes the Asian honey bee (*A. ceranae*) and the European honey bee [6,7,8]. *Nosema ceranae* is cited as one of the significant causes of bee colony death, infecting the epithelial cells of the bee’s midgut, and inducing oxidative and energetic stress, which further worsens the fitness and health of bee colonies [9,10]. 

The most common group of pesticides are synthetic pyrethroids, with a wide range of over 1000 insecticides used in insect control [11,12,13]. Although based on the chemical structure and biological activity of pyrethrum (an extract of plants from the *Chrysanthemum* genus), later development of synthetic pyrethroids involved extensive chemical modifications to improve the formulations [14,15]. Initially, pyrethroids prevent the insects from maintaining the body’s normal position, after which they die or recover. Pyrethroids can repel insects or stop food intake [16,17,18]. These pesticides are commonly used in mosquito control in agricultural and urban sites [13]. In certain areas, resistance of some mosquitoes to pyrethroids has been observed; thus, a combination of deltamethrin with piperonyl butoxide is applied [13,17]. Deltamethrin and piperonyl butoxide are two synthetic pyrethroids that are synergistic when combined. Pyrethroids affect the feeding behavior of insects, including bees [19]. Affected bees die outside the hive, reducing the possibility of further hive contamination, while the brood remains inaccessible to insecticide action [20]. It was found that deltamethrin exerts neural effects in bees, by interfering with homing behavior and inducing an exacerbated phototropism in the foragers [21]. Dworzanska et al. (2020) explained different levels of bees’ sensitivity to deltamethrin by mechanisms that can metabolize the toxin in the absence of other stressors that weaken bees and harm the bee colony. Deltamethrin can change honey bee tissues, primarily the intestines, thereby impairing enzyme activity, memory ability, and behavioral patterns. Deltamethrin also induces reproductive problems related to the viability of sperm and disorders of the ovaries [20,22]. 

Electron Paramagnetic Resonance (EPR) spectroscopy is indispensable in our research, particularly for the following:

1. Investigating the mechanisms of oxidative stress;
2. Specifically detecting paramagnetic species such as reactive oxygen species (ROS);
3. High spectral resolution;
4. Time-resolved studies of kinetic processes. When the organism’s antioxidative defenses against ROS are not sufficiently effective, the result is oxidative and energetic stress, an imbalance between oxidative and antioxidative species. Although ROS are crucial messengers to immune cells and accomplish other essential tasks, they can kill too many host cells by reacting with many molecules, such as DNA and RNA, and cell structures such as lipid membranes [23]. When needed, antioxidant enzymes combat ROS. The most frequently used markers of oxidative stress in bee research are the concentration of malondialdehyde (MDA) and the activity of the antioxidative enzymes: catalase (CAT), glutathione S-transferase (GST), and superoxide dismutase (SOD) [24,25,26,27,28].

The potential of *Agaricus bisporus* mushroom extract to influence the expression of bees’ immune-related genes [25] led us to investigate its protective potential in combating pesticide/nosema negative impact. In this study, we tested *A. bisporus* protective potential in bees infected with *N. ceranae* and exposed to deltamethrin and piperonyl butoxide as a synergistic component.

## 2. Materials and Methods

### 2.1. The Tested Preparations 

#### 2.1.1. Agaricus Bisporus Extract

The extract from a commercially grown white button mushroom (*Agaricus bisporus*, strain A15) was acquired using an aqueous extraction method. Following the protocol by [25,29] Klaus et al. [29] and Glavinic et al. [25], dried and powdered mushroom fruit bodies were extracted with distilled water at 121 °C and 1.2 bar for 60 min. The resultant solution was filtered, evaporated to one-third of its original volume, and precipitated with 96% ethanol overnight. After precipitation, the extract was centrifuged, dried at 40 °C, and ground into a fine powder. For experimental consistency and based on prior effective dosages identified in bee health research, the concentration of the extract was standardized at 200 µg/g in 30% (*w*/*v*) sucrose solution [25]. This concentration was chosen for its previously documented efficacy in enhancing the survival and health parameters of honey bees infected with *N. ceranae*, as well as its minimal toxicity profile [25,30]. The processed extract was stored in a refrigerator at 4 °C until further use.

#### 2.1.2. Pesticides

Deltamethrin (K Othrine^®^ WG250, Monheim am Rhein, Germany) and the combination of deltamethrin with piperonyl butoxide (K Obiol^®^ EC25, Monheim am Rhein, Germany). Pesticide solutions were prepared in a 30% solution of water–sugar (sucrose) syrup. The deltamethrin concentration of 500 µg/kg corresponds to the field realistic (found in rapeseed pollen after the pesticide treatment) concentration [31]. 

### 2.2. Bees and the Experimental Design

In June 2023, brood frames were carefully taken from three different honey bee colonies at the apiary of the University of Belgrade, Faculty of Veterinary Medicine. These frames were then placed in an incubator and kept at a temperature of 34 ± 1 °C and a humidity level of 66 ± 1%. To ensure that hatched bees did not escape, frames were placed into the net bags.

On the day of emerging, worker bees were gently removed from the frames and randomly distributed into cages, with each cage holding exactly 60 bees. The experiment included 12 groups (Table 1), each containing 3 cages as replicates, to ensure reliable experimental results. The groups were established to study the impact of treatments on bee health and survival. These treatments included a control group with no treatment *(NT),* groups infected with *N. ceranae (N)*, and groups treated with *A. bisporus* extract *(Ab)*, those treated with *Deltamethrin (D),* and those treated with both *Deltamethrin* and *Piperonyl butoxide (DPb)*, as well as different combination of listed factors (pesticides, *Nosema* and extract) including 12 different groups in total (Table 1).

All the bee groups were provided with a syrup solution containing 30% sucrose throughout the 14-day experiment. The syrup was mixed with the *Nosema* spores/pesticides/*A. bisporus* extract according to the experimental design (Table 1). The consumption of this syrup was monitored daily by measuring the amount of applied syrup and the remaining amount of syrup after feeding within 24 h [32]. Daily mortality rates were recorded by counting and removing dead bees from each cage.

### 2.3. Inoculum Preparation and Experimental Infection

Experimental infection of bees and inoculum preparation followed previously described procedures [24,33]. Briefly, the abdomens of bees infected with *N. ceranae* were crushed in water to create the inoculum. Using Cantwell’s method, the number of spores was calculated [34]. A freshly made spore suspension with 99% vitality (assessed with 4% trypan blue) (Sigma-Aldrich, Steinheim, Germany) was diluted with 30% sucrose solution to achieve a final 1 × 10^6^ spores/mL concentration. Artificial infection with spores of *N. ceranae* was carried out on the third day in bees from the infected control group (N) and other Nosema-infected groups (N-AB, D-N, DPb-N, D-N-Ab, and DPb-N-Ab).

### 2.4. Bee Sampling 

On days 7 and 14, we sampled 20 bees (10 bees for counting N. ceranae spores and 10 bees to measure oxidative stress levels) from each cage (with 60 bees); thus, 40 bees in total were sampled from each cage. The remaining 20 bees in each cage were observed continuously throughout the experiment to determine survival rates. 

### 2.5. Nosema Spore Counting 

On days 7 and 14, 10 bees from each cage were chosen for analysis. The bees’ abdomens were crushed in 1 mL of water using a Tissue Lyser II and tungsten carbide beads (QIAGEN, Hilden, Germany). This method guaranteed tissue disruption, releasing the spores. The resulting mixture was then inspected under a microscope using a hemocytometer to accurately measure *N. ceranae* spores. The spore-counting process followed the procedures detailed by Cantwell (1970). The guidelines set by the World Organisation for Animal Health [35] ensure the assessment of spore levels in each bee sampled.

### 2.6. Antioxidant Activity of Tested Preparations

#### 2.6.1. Reagents and General Experimental Procedures

For these analyses, 30% hydrogen peroxide—H_2_O_2_ (Lach-Ner, Neratovice, Czech Republic), Iron (II) sulfate heptahydrate and ethanol (Merck KGa, Germany), AccuGENE deionized 18 MΩ water (Lonza, Bornem, Belgium), and 5-(Diethoxyphosphoryl)-5-methyl-1-pyrroline-N-oxide—DEPMPO (Focus Biomolecules, USA), and Methanol (POCH, Poland) were used. All EPR spectra were recorded on a Bruker ELEXSYS-II E540 spectrometer (Bruker, Berlin, Germany) operating in X-band mode, by inserting the samples into the gas-permeable Teflon tubes (Zeus Industries Inc., Largo, FL, USA)

#### 2.6.2. Determination of the Scavenging Activity Toward the DPPH Radicals

The 2,2-diphenyl-1-picrylhydrazyl (DPPH) serves as a stable free radical that is easily obtainable and commonly employed in evaluating the antioxidant capabilities of various substances, notably through UV-Vis spectroscopy. Conventional DPPH assays involve the reduction of its violet ethanol solution from its free radical state to a colorless or yellowish form in the presence of antioxidant compounds [36]. Conversely, the DPPH radical is suitable for Electron Paramagnetic Resonance (EPR) assays, as its radical state is EPR active while the reduced state is EPR silent. EPR methods are preferred, particularly because they are not reliant on the optical properties of the system being studied. The distinct EPR signal of the DPPH radical enables the determination of the initial radical concentration and the efficacy of *A. bisporus* extract and pesticide solutions in reducing its presence within the system [37].

Samples were studied by adapting the method of [37,38] to detect the activity of the aqueous solutions of pesticide and *A. bisporus* extract toward the DPPH radicals. Briefly, 1 μL of the sample was added to 29 μL of the DPPH solution in water (final concentration 7 μM). This mixture was transferred into the gas-permeable Teflon tube, and the EPR signal was recorded at 2 min under the following settings: center field 3500 G, microwave power 10 mW, microwave frequency 9.85 GHz, modulation frequency 100 kHz, and modulation amplitude 2 G. Control recordings used samples with the same volume of water.

#### 2.6.3. Determination of the Scavenging Activity Toward the •OH Radicals

To quantify the concentration of hydroxyl (•OH) radicals in the system, an aqueous solution of pesticides and *A. bisporus* extract alongside a Fenton reaction mixture was prepared containing the spin-trap 5-(Diethoxyphosphoryl)-5-methyl-1-pyrroline-N-oxide (DEPMPO) [39,40]. DEPMPO was chosen due to its recognized selectivity and the extended half-life of the DEPMPO/OH spin adduct (132 min [41]). To mitigate the impact of natural degradation of the spin adduct, it was crucial to promptly acquire EPR spectra immediately following the initiation of the Fenton reaction. In brief, 30 µL of the sample, which contained 25 µL of water, 1µL of pesticide solution or *A. bisporus* extract, 2 µL of H_2_O_2_ (final concentration of 0.35 mM), and 1 µL of DEPMPO (final concentration of 3.5 mM) was transferred into the gas-permeable Teflon tube and 1 µL of FeSO_4_ (final concentration 0.15 mM) was applied just before the EPR spectra were acquired. Recordings were made using the following experimental settings: center field 3500 G, microwave power 10 mW, microwave frequency 9.85 GHz, modulation frequency 100 kHz, and modulation amplitude 1 G. A control recording was conducted by replacing 1µL of pesticide solution or *A. bisporus* extract with an equal volume of water.

### 2.7. Analyses of Oxidative Stress Parameters in Bees

The spectrophotometric analyses described in Dubovskiy et al. (2008) [42] and modified by Glavinic et al. [24,26] were used to measure these oxidative stress parameters: activities of the antioxidative enzymes superoxide dismutase (SOD), catalase (CAT) and glutathione S-transferase (GST), and the concentrations of malondialdehyde (MDA). On days day 7 and 14, pools of 10 bees collected from each cage were analyzed on a UV/VIS Spectrophotometer BK-36 S390. The MDA concentration is expressed in nmol/mg of protein, and the specific activities of the enzymes are expressed in units of activity per mg of protein (U/mg of protein).

### 2.8. Statistical Analyses 

The data were statistically analyzed using SPSS Statistics 29.0 software (IBM^®^, Armonk, NY, USA), and the figures were designed using GraphPad Prism 10.0 (GraphPad Software Inc., Boston, MA, USA). In the first step, the normality of the obtained data was examined using the Shapiro–Wilk test. The results for the number of spores of *N. ceranae* have a normal distribution (*p* > 0.05), while the data related to oxidative stress deviated from the normal distribution (*p* < 0.05). Therefore, analysis of variance (ANOVA) was performed to determine the effect of treatment, exposure time (7 and 14 days), and treatment × time interaction on the number of spores of *N. ceranae*. Subsequently, a post hoc test (Tukey) was used to determine significant differences between pairs of average characters evaluated between treatments. On the other hand, to test the hypothesis on the equality of medians among three or more groups as well as at the two-time points of sampling, the Kruskal–Wallis test was utilized for oxidative stress parameters. In addition, the significance of the difference between two groups at the same time point was ascertained using the Mann–Whitney U test, while the Wilcoxon test was used to investigate differences between the two time points of sampling within the same group of bees. The survival of bees was tested using the log-rank test, which was graphically presented as the Kaplan–Meier survival curve. Significance was declared at *p* < 0.05, *p* < 0.01, and *p* < 0.001, and a tendency was acknowledged at 0.05 ≤ *p* < 0.10.

## 3. Results

### 3.1. Bee Survival 

When simultaneously analyzing the number of dead bees in the control and all treated groups, the log-rank test shows significant differences (X^2^ = 19.90; *p* = 0.047). 

**Figure 1:**
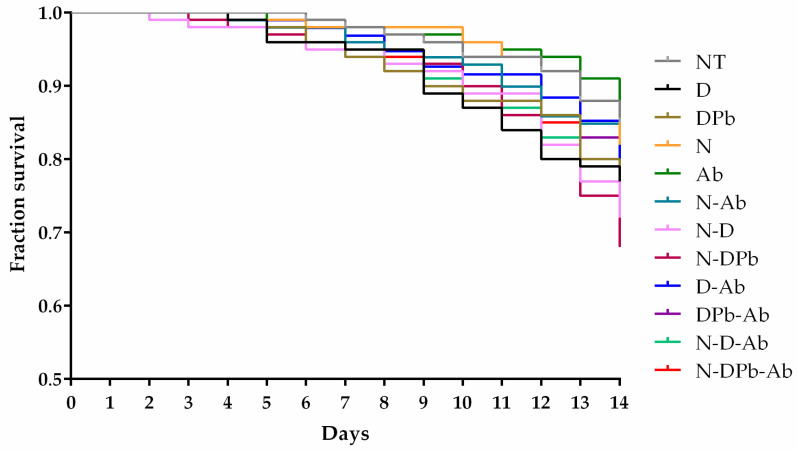
Survival rate of bees across control and various treated groups over a 14-day experimental period. The Kaplan–Meier survival curve demonstrates significant differences in mortality rates, with the highest mortality observed in the N-DPb group, followed by the N-D group. The Ab group, treated solely with *Agaricus bisporus* extract, shows the lowest mortality rate. Significance values are included to compare differences between groups. The group labels are described in Table 1.

The highest mortality rate was recorded in the N-DPb group, which was significantly higher than the NT (*p* = 0.004), N (*p* = 0.018), Ab (*p* = 0.006), and N-Ab (*p* = 0.022) groups. The N-D group also exhibited a high mortality rate, significantly higher compared to the NT (*p* = 0.023) and Ab (*p* = 0.004) groups, and tended to be higher compared to N (*p* = 0.075) and N-Ab (*p* = 0.082) groups (Figure 1). Although the Ab group showed the lowest mortality, the statistical significances were detected compared to D (*p* = 0.034), N-DPb (*p* = 0.006), D-Ab (*p* = 0.011), N-D-Ab (*p* = 0.024), and N-DPb-Ab (*p* = 0.016) groups. In addition, the mortality rate in the Ab group tended to be lower than in the DPb (*p* = 0.075) and DPb-Ab (*p* = 0.080) groups of bees.

### 3.2. Number of Nosema Spores 

**Figure 2:**
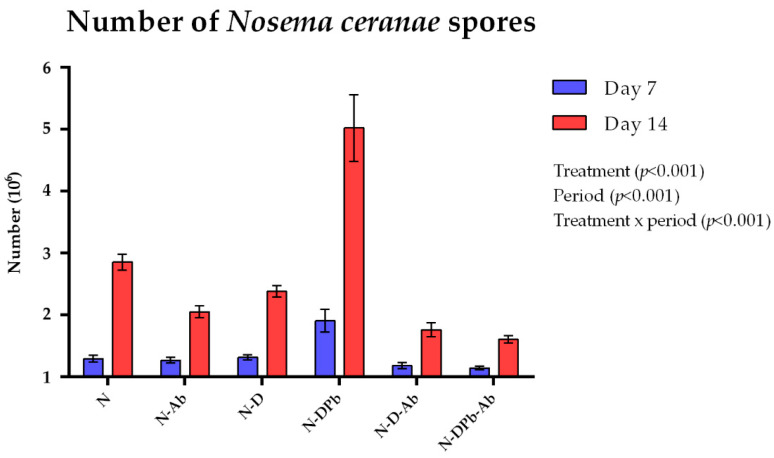
Mean spore counts of *Nosema ceranae* in bees from different experimental groups on days 7 and 14. The treatment groups are described in Table 1.

Samples from the following non-treated control (NT) and non-infected groups: deltamethrin (D), deltamethrin/piperonyle butoxide (DPb), *Agaricus bisporus* (Ab), deltamethrin–*Agaricus bisporus* (D-Ab), and deltamethrin/Piperonyle butoxide–*Agaricus bisporus* (DPb-Ab), collected on day 7 and day 14 remained negative for *N. ceranae* spores. Analyzing positive samples (Figure 2), the ANOVA test showed significant differences in the numbers of *N. ceranae* spores on both sampling days (7 and 14), comparing all groups together (*p* < 0.001). Tukey’s test of pairwise comparisons revealed a significantly higher number of spores on day 14 compared to day 7, when all groups were analyzed (Figure 1). Both on days 7 and 14, a significantly higher number of spores was recorded in the N-DPb group compared to all other groups (*p* < 0.05). According to Tukey’s test, on day 7, the spore loads were significantly lower (*p* < 0.05) in N-DPb-Ab compared to the N-DPb, N-D, N-Ab, and N groups. At the end of the experiment (day 14), the spore loads were significantly lower (*p* < 0.05) in N-DPb-Ab compared to N, N-Ab, N-D, and N-DPb groups.

### 3.3. Antioxidant Activity of Testing Solutions

#### 3.3.1. Scavenging Activity Toward DPPH Radicals

**Figure 3:**
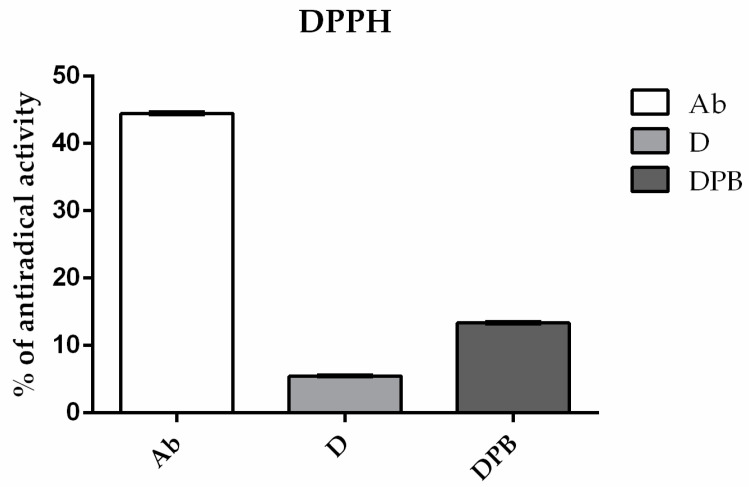
Percentage of DPPH radical scavenging activity of the aqueous *Agaricus bisporus* extract (Ab), deltamethrin (D), and the combination of deltamethrin and piperonyl butoxide (DPb).

The analysis of the antioxidant activity of the tested preparations (normalized against a control set at 100%), which were consumed by bees in 30% aqueous sugar syrup, revealed notable distinctions among the groups (*p* < 0.5). The Ab sample, a water extract of *Agaricus bisporus*, stood out with significant antioxidant activity, which is clearly illustrated in Figure 3. It is shown that the extract not only effectively reduces the intensity of the DPPH radicals EPR signals but also shows superiority over other tested solutions. 

#### 3.3.2. Scavenging Activity Toward •OH Radicals

**Figure 4:**
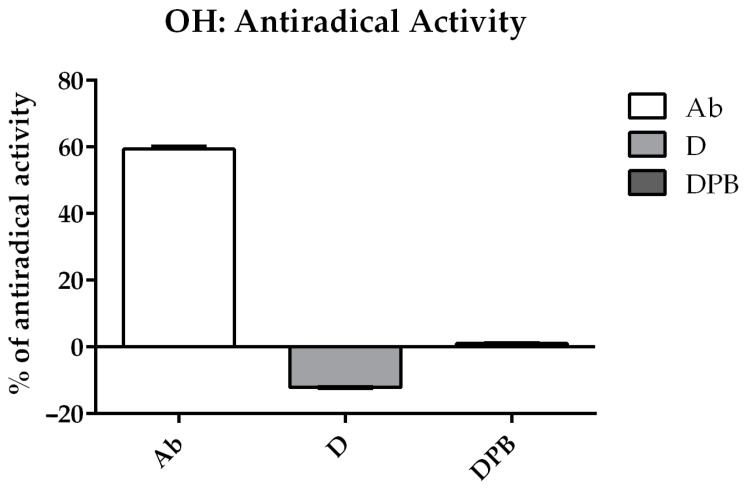
Percentage of hydroxyl radical (•OH) scavenging activity of aqueous extract of *Agaricus bisporus* (Ab), deltamethrin (D), and deltamethrin with piperonyl butoxide (DPb).

**Figure 5:**
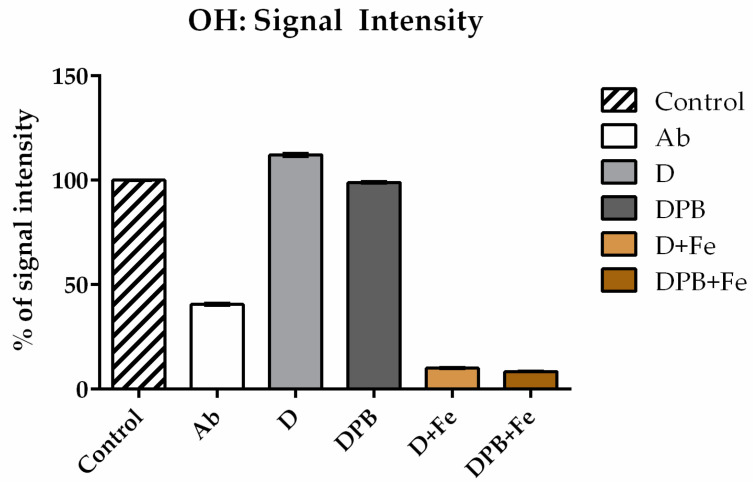
Electron Paramagnetic Resonance (EPR) signal intensity indicating •OH radical generation in reaction with test solutions: *Agaricus bisporus* extract (Ab), deltamethrin (D), and deltamethrin with piperonyl butoxide (DPb).

The water extract of *Agaricus bisporus* demonstrated remarkable efficiency in removing •OH radicals, as shown in Figure 4. In contrast, the D solution (deltamethrin) not only was ineffective in removing •OH radicals but actually contributed to their production. This phenomenon was diminished with the addition of piperonyl butoxide (testing DPb solution), but effective radical scavenging was still not achieved. To investigate whether deltamethrin interacts with H_2_O_2_ or Fe^2+^ (constituents of the Fenton reaction which leads to the formation of •OH radicals), deltamethrin (and, afterward, DPb) was introduced into systems containing either H_2_O_2_ or Fe^2+^ along with the spin-trap DEPMPO. While no EPR signal of DEPMPO/OH adduct was detected in combination with H_2_O_2_, this signal was observed when D and DPb were combined with Fe^2+^ (Figure 5). On the other hand, the Ab (*A. bisporus*) extract effectively scavenges hydroxyl radicals, outperforming the detrimental effects of D and DPb.

Here, we show the interaction of the test solutions with H_2_O_2_ or Fe^2+^ (components of the Fenton reaction) in the presence of the spin-trap DEPMPO.

### 3.4. Results of Oxidative Stress Parameters in Bees

#### 3.4.1. Catalase Activity

**Figure 6:**
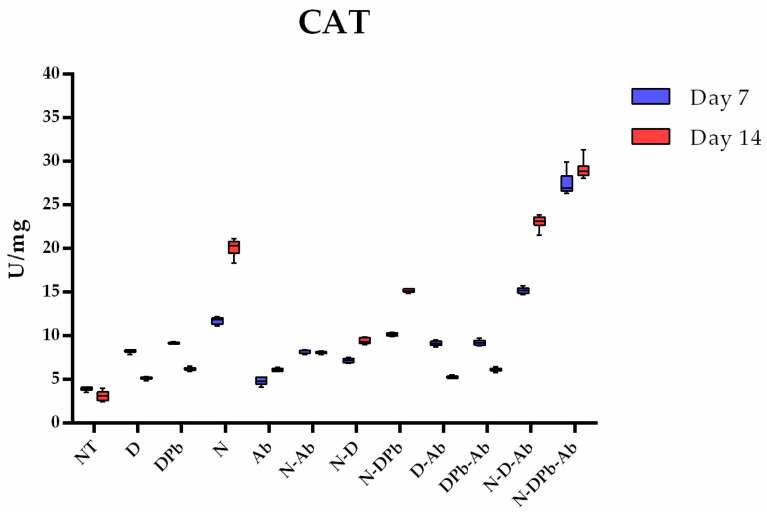
CAT activity. Catalase (CAT) activity in the experimental groups on days 7 and 14. Groups and group labels are listed in Table 1. *p*-values of pairwise comparison are presented in Supplementary Tables S1 (for day 7) and S2 (for day 14).

According to the Kruskal–Wallis test, there were significant differences (*p* < 0.001) in CAT activity between all the studied groups (Figure 6). On day 7, the N-DPb-Ab had a significantly higher (*p* < 0.05), while the NT group had significantly lower (*p* < 0.05) CAT activity compared to all other groups. From day 7 to day 14, CAT activity increased significantly in groups N, N-D, N-DPb, and N-D-Ab. At the end of the study (day 14), the N-DPb-Ab group maintained the highest level, while the NT group maintained the lowest level of CAT activity compared to all other groups (*p* < 0.05). Individual *p*-values of pairwise comparison are presented in Supplementary Tables S1 (for day 7) and S2 (for day 14). 

#### 3.4.2. Superoxide Dismutase Activity

**Figure 7:**
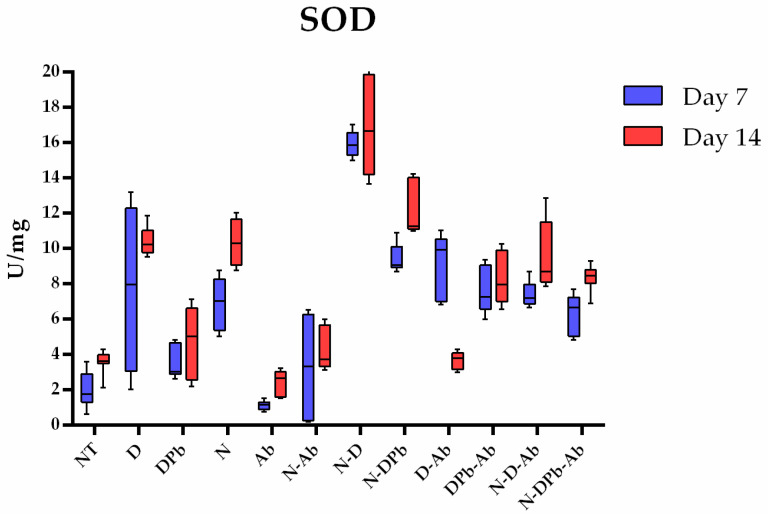
SOD activity. Superoxide dismutase (SOD) activity in the experimental groups on days 7 and 14. Groups and group labels are listed in Table 1. *p*-values of pairwise comparison are presented in Supplementary Tables S3 (for day 7) and S4 (for day 14).

According to the Kruskal–Wallis test, there were significant differences (*p* < 0.001) in SOD activity between all groups (Figure 7). On day 7, the N-D group had significantly higher (*p* < 0.05), while the Ab group had significantly lower (*p* < 0.05) SOD activity compared to all other groups. From day 7 to day 14, SOD activity increased significantly in most of the groups with the exemption of groups D, N-Ab, and DPb-Ab, which had a significantly lower SOD activity on day 14 compared to day 7. However, at the end of the study (day 14), the N-D group maintained the highest level, and the Ab group maintained the lowest level of SOD activity compared to all other groups (*p* < 0.05). Individual *p*-values of pairwise comparison are presented in Supplementary Tables S3 (for day 7) and S4 (for day 14).

#### 3.4.3. Glutathione S-Transferase Activity

**Figure 8:**
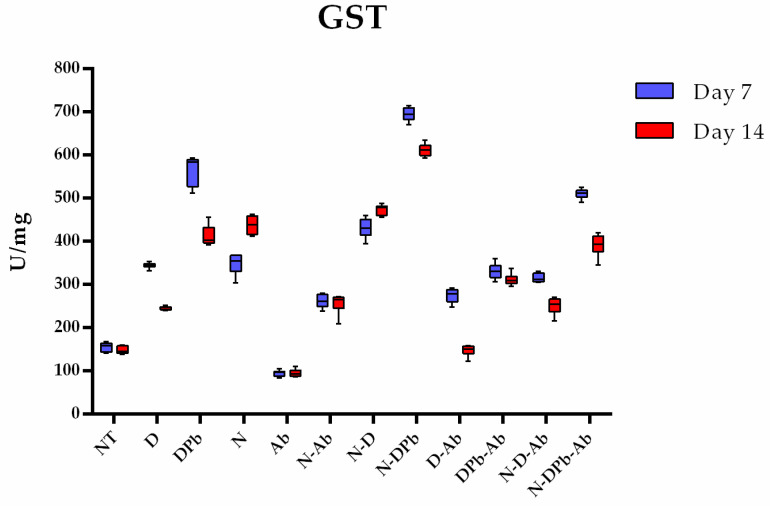
GST activity. Glutathione S-transferase (GST) activity in the experimental groups on days 7 and 14. Groups and group labels are listed in Table 1. *p*-values of pairwise comparison are presented in Supplementary Tables S5 (for day 7) and S6 (for day 14).

According to the Kruskal–Wallis test, there were significant differences (*p* < 0.001) in GST activity between the experimental groups (Figure 8). On day 7, the N-DPb group had a significantly higher (*p* < 0.05), while the Ab group had significantly lower (*p* < 0.05) GST activity compared to all other groups. From day 7 to day 14, GST activity significantly increased only in the N group, while in the other groups (*p* < 0.05), GST levels were significantly lower (*p* < 0.05) on day 14. Despite a decrease from day 7, the GST level on day 14 was significantly higher in the N-DPb group compared to all other groups (*p* < 0.05), whereas the lowest GST activity on day 14 was observed in the Ab group compared to all other groups (*p* < 0.05). Individual *p*-values of pairwise comparison are presented in Supplementary Tables S5 (for day 7) and S6 (for day 14). 

#### 3.4.4. Malondialdehyde Concentrations

**Figure 9:**
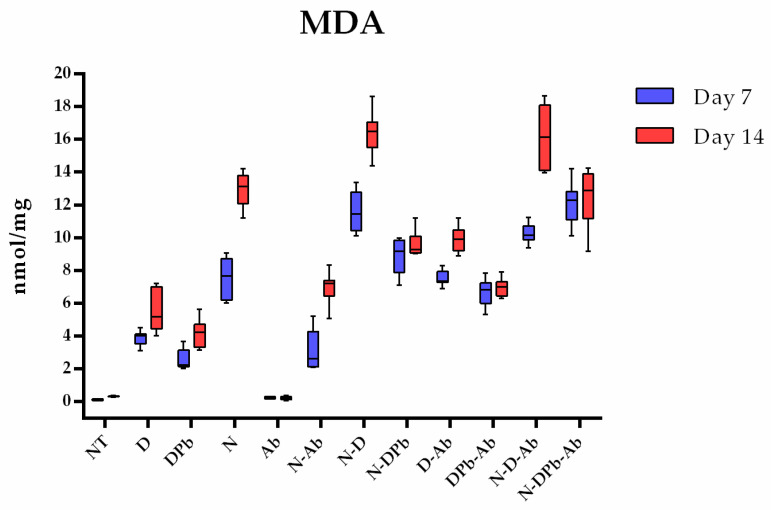
MDA concentrations. Malondialdehyde (MDA) concentrations (nmol/mg of total proteins) in the experimental groups. Groups and group labels are listed in Table 1. *p*-values of pairwise comparison are presented in Supplementary Tables S7 (for day 7) and S8 (for day 14).

According to the Kruskal–Wallis test, there were significant differences (*p* < 0.001) in MDA concentrations between the groups studied (Figure 9). On day 7, the N-D group had significantly higher (*p* < 0.05) MDA concentrations compared to all other groups, except the N-DPb-Ab group, while the NT and AB groups had significantly lower (*p* < 0.05) MDA concentrations compared to all other groups. The comparison of MDA concentrations between the two sampling times revealed a significant (*p* < 0.05) difference in concentrations, which were higher on the 14th day in the groups N, N-Ab, N-D, D-Ab, and N-D-Ab. Despite the N-D group exhibiting significantly higher concentrations of MDA on day 7 compared to other groups except N-DPb-Ab, on day 14 of the study, the highest concentration of MDA was observed in the N-D-Ab group. (*p* < 0.05). Individual *p*-values of pairwise comparison are presented in Supplementary Tables S7 (for day 7) and S8 (for day 14). 

## 4. Discussion

This study clearly illustrates multi-factorial relationships between the bees and combined chemical treatments and/or *Nosema* infection. This became very obvious when deltamethrin was applied together with piperonyl butoxide and *N. ceranae* infection. Recent studies have demonstrated that several supplements and extracts benefit the well-being of bees affected by *N. ceranae* infections [24,43,44,45]. The administration of *A. bisporus* mushroom extract aided the survival of bees infected with spores of *N. ceranae*, as seen in the study conducted by [46]. No difference in statistical significance in the mortality rate of bees between the untreated group (NT) and the group that received a sugar solution containing extract *A. bisporus* (Ab); moreover, this supplementation aids bee survival (Figure 1). The lack of *Nosema* spores in non-infected bee groups (NT, D, DPb, Ab, D-Ab, and DPb-Ab) confirms that the cage-type experiment used in this study prevented cross-contamination [33]. The detection of *Nosema* spores in all infected groups confirmed that the inoculum, with a final concentration of 1 × 10^6^ spores/mL, effectively induced the infection, as shown in earlier research [24,25]. On days 7 and 14 of the experiment, the groups infected with *N. ceranae* spores and treated with deltamethrin and/or piperonyl butoxide (N, N-D, N-DPb) showed the highest spore load (Figure 2), while lower *Nosema* load was in the group treated with *Agaricus* mushroom extract. This finding confirms that *A. bisporus* has an anti-*Nosema* effect [46]. The N-DPb group showed a significantly higher spore load on days 7 and 14 than the N-D group, suggesting that piperonyl butoxide acts synergistically with deltamethrin [47], increasing the spore load at the end of the experiment. Brattsten (1988) suggests that piperonyl butoxide interferes with the cytochrome P450 and esterase pathways, which are responsible for detoxifying and eliminating toxins, by inhibiting them [48]. This inhibition and retention of hazardous substances could be a reason why pesticides (even at lower concentrations) exert a synergistic effect with piperonyl butoxide, as was shown in several insect species [49,50].

**Figure 10:**
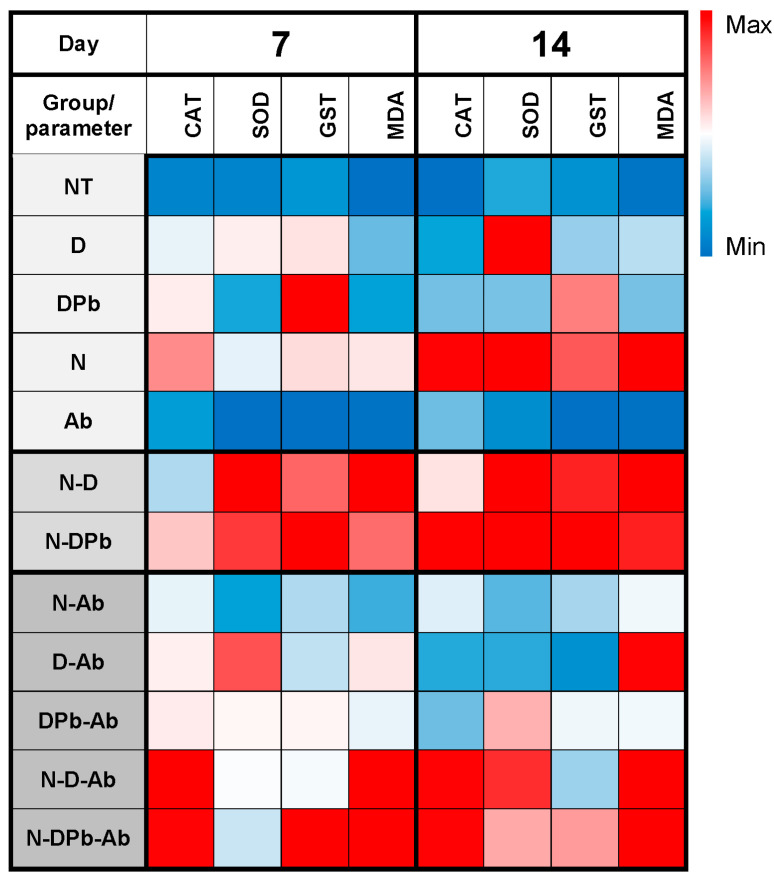
Antioxidant enzyme (CAT, SOD, GST) activity and MDA concentration. Heat map of median values for the activity of catalase (CAT), superoxide dismutase (SOD), glutathione S-transferase (GST), and concentration of malondialdehyde (MDA) in honey bees from experimental groups. Groups and group labels are listed in Table 1. *p*-values of pairwise comparison are presented in Supplementary Tables S1–S8.

Having in mind that the sugar solution impacts the antioxidant performance in testing bioactive substances in EPR [51], we decided to test extract in sugar-free aqueous solutions to mitigate the potential confounding effects of the matrix (sucrose and its impurities) on the scavenging activity of the selected compounds. Previous studies have confirmed that deltamethrin induces oxidative stress and exhibits high cytotoxicity toward living organisms [52,53,54]. Notably, deltamethrin not only fails to scavenge free radicals but also actively contributes to the generation of reactive oxygen species [55,56]. To investigate whether deltamethrin interacts with H_2_O_2_ or Fe^2+^ (constituents of the Fenton reaction, which leads to the formation of •OH radicals), deltamethrin (and, afterward, deltamethrin in combination with piperonyl butoxide—DPb) was introduced into systems containing either H_2_O_2_ or Fe^2+^ along with the spin-trap DEPMPO. While no EPR signal of DEPMPO/OH adduct was detected in combination with H_2_O_2_, this signal was observed when deltamethrin (D) and deltamethrin and piperonyl butoxide (DPb) were combined with Fe^2+^ (Figure 5). These findings may contribute to understanding how deltamethrin disrupts iron metabolism in animal models [57]. On the other hand, the *A. bisporus* (Ab) extract effectively scavenges hydroxyl radicals, outperforming the detrimental effects of D and DPb. This underlines the exceptional antioxidant capabilities of *A. bisporus*, positioning it as a safe, natural extract that could have a great role in antioxidative protection and improvement of bee health. In this study, bee mortality (Figure 1) and *Nosema* levels (Figure 2) were significantly decreased in groups treated with *A. bisporus* extract. These findings suggest that mushroom extract can improve the health of the bees combating *Nosema* infection. Moreover, the activity of SOD, CAT, and GST and the concentration of MDA were lower in groups fed with the addition of *A. bisporus* extract in the majority of the groups (Figure 10), confirming the extract’s positive impact on antioxidative protection. 

In this study, deltamethrin, known to induce oxidative stress in bee populations [58], also induced such stress recorded through higher levels of monitored oxidative stress-related parameters. Interestingly, a combination of deltamethrin and piperonyl butoxide induced lower oxidative stress than deltamethrin on its own, according to the results of the activity of monitored enzymes. Furthermore, our findings (EPR analyses) showed that D and DPb induce the generation of •OH radicals. However, further investigation is needed to fully elucidate the underlying mechanism [59]. 

Experimental groups that were infected with spores of *N. ceranae* (N, N-D, N-DPb, N-D-Ab, and N-DPb-Ab) showed increased CAT activity on day 14, compared to the other groups, indicating that *N. ceranae* infection was a significant inducer of oxidative stress. An exception is the experimental group infected with *N. ceranae* and treated with *A. bisporus* extract (N-Ab), in which CAT activity was lower on day 14, which could be attributed to the mushroom extract protective effect. SOD activity also revealed a positive effect of the mushroom extract in the reduction of oxidative stress induced by *Nosema* (visible when comparing N and N-Ab groups), as well as deltamethrin (D/D-Ab groups) on day 14. Moreover, the experimental group treated with these two stressors (*Nosema* and deltamethrin: N-D) exhibited the highest oxidative stress (SOD activity was significantly higher than all other groups) on both days 7 and 14. However, the addition of *A. bisporus* extract (monitored through the N-D-Ab group) reduced this oxidative stress, which is also noticeable through SOD activity (Figure 7). These positive antioxidant effects could be attributed to several compounds of a mushroom extract, which are known to have antioxidant characteristics (polysaccharides, ergothioneine, and seleniu) [60,61]. These agents might contribute to the reduction of oxidative stress markers, acting as neutralizers of reactive species [61].

Analyzing the MDA concentration, the *A. bisporus* (Ab) group showed the lowest value on both days 7 and 14. However, in combination with pesticides, *A*. *bisporus* (groups D-Ab and DPb-Ab) could not significantly affect the reduction of MDA concentration. The reason for this may be prolonged exposure to pesticides, which can result in persistent cellular damage, rendering antioxidants ineffective [62,63]. 

The natural mushroom extract’s effect in reducing the harm of environmental chemicals in this study is more significant considering growing worries about general bee health and bee population sustainability. The ramifications of pesticide usage become evident while minimizing the side effects in order to reduce negative impacts on pollinators presents one of the most important measures [64,65]. Future studies should precisely target the mechanisms through which *A. bisporus* brings health benefits to honey bees [25]. Natural-based treatments in beekeeping have a long-term effect on bee colonies because they secure pollination and biodiversity as well as obtaining safe honey bee products. Research in which multiple stressors (pesticides, biological pathogens, and food supplements like *A. bisporus*) are involved is of great importance, as it simulates real field conditions. 

In the future, it would be beneficial to investigate ways to use supplements such as *A. bisporus* extract to promote bee well-being and help them cope with stress. Furthermore, examining how various management techniques can work together might result in eco-beekeeping methods, which could play a vital role in protecting bee populations that are essential for pollination and biodiversity preservation.

## 5. Conclusions

This research explored how the combination of deltamethrin, piperonyl butoxide, *N. ceranae* infection, and *A. bisporus* supplementation affects stress in honey bees. The *A. bisporus* (Ab) extract effectively scavenges DPPH and hydroxyl radicals, surpassing the scavenging capabilities of D and DPb. Furthermore, EPR analyses revealed that D and DPb even induce the generation of hydroxyl radicals, leading to potential detrimental effects.

Our findings reveal differences in how bees respond to combined treatments, such as complex interactions between chemical stressors, biological pathogens, and dietary supplements. Thus, our results indicate that deltamethrin, as well as its combination with piperonyl butoxide, induced oxidative stress in monitored bees. *A. bisporus* extract has shown antioxidant properties in bees affected with both *N. ceranae* and pesticides. However, adding *A. bisporus* to the bee diet did not reduce stress when bees were faced with multiple stressors. These findings have implications for beekeeping and environmental science, emphasizing the importance of pesticides and pathogen management approaches, as well as the potential of natural-based supplementation.

## Figures and Tables

**Table 1:** Experimental design.

Groups ^1^	*Nosema ceranae* ^2^	Deltamethrin ^2^	Deltamethrin and Piperonyl Butoxide ^2^	*Agaricus bisporus* ^2^
NT	Non-treated group			
D		3		
DPb			3	
N	3			
Ab				1
N-Ab	3			1
N-D	3	3		
N-DPb	3		3	
D-Ab		3		1
DPb-Ab			3	1
N-D-Ab	3	3		1
N-DPb-Ab	3		3	1

^1^ The group names/labels were determined according to abbreviations of the treatment/infection: deltamethrin (D), piperonyl butoxide (Pb), *Agaricus bisporus* (Ab), and *Nosema ceranae* (N), or non-treated (NT) group. ^2^ Day on which (after emergence) the treatment/infection was performed.

## Data Availability

The data presented in this study are available on request from the corresponding author.

## Supplementary Materials

The following supporting information can be downloaded at: https://www.mdpi.com/article/10.3390/life14111498/s1, Table S1: CAT (day 7). Table S2: CAT (day 14). Table S3: SOD (day 7). Table S4: SOD (day 14). Table S5: GST (day 7). Table S6: GST (day 14). Table S7: MDA (day 7). Table S8: MDA (day 14).
