# Supplementary material for: The Potential of Agaricus bisporus in Mitigating Pesticide-Induced Oxidative Stress in Honey Bees Infected with Nosema ceranae
Source: Life (Basel). 2024 Nov 17;14(11):1498. doi: 10.3390/life14111498 (PMC11595567; doi:10.3390/life14111498)
Supplement: Supplementary file 1 [file life-14-01498-s001.zip › life-3267291-supplementary.pdf]

Supplementary

S1 – CAT (day 7)

**P values derived from Mann Whitney U test for day 7 of the experiment**												
	NT	D	DPb	N	Ab	NAb	ND	NDPb	DAb	DPbAb	NDAb	NDPbAb
NT												
D	<0.001											
DPb	<0.001	<0.001										
N	<0.001	<0.001	<0.001									
Ab	<0.001	<0.001	<0.001	<0.001								
NAb	<0.001	>0.9999	<0.001	<0.001	<0.001							
ND	<0.001	<0.001	<0.001	<0.001	<0.001	<0.001						
NDPb	<0.001	0.0040	0.0036	<0.001	<0.001	0.0037	0.0010					
DAb	<0.001	<0.001	0.9486	<0.001	<0.001	<0.001	<0.001	0.0132				
DPbAb	<0.001	<0.001	0.7125	<0.001	<0.001	<0.001	<0.001	0.0040	0.9812			
NDAb	<0.001	<0.001	0.0007	0.0037	<0.001	<0.001	<0.001	0.0028	0.0004	0.0005		
NDPbAb	<0.001	<0.001	<0.001	<0.001	<0.001	<0.001	<0.001	<0.001	<0.001	<0.001	0.0005	

S2 – CAT (day 14)

**P values derived from Mann Whitney U test for day 14 of the experiment**												
	NT	D	DPb	N	Ab	NAb	ND	NDPb	DAb	DPbAb	NDAb	NDPbAb
NT												
D	<0.001											
DPb	<0.001	<0.001										
N	<0.001	<0.001	<0.001									
Ab	<0.001	<0.001	0.3073	<0.001								
NAb	<0.001	<0.001	<0.001	<0.001	<0.001							
ND	<0.001	<0.001	<0.001	<0.001	<0.001	<0.001						
NDPb	<0.001	<0.001	<0.001	<0.001	<0.001	<0.001	0.0006					
DAb	<0.001	0.1683	0.0040	<0.001	0.0036	0.0040	0.0055	0.0012				
DPbAb	<0.001	0.0002	0.2136	<0.001	0.7796	<0.001	<0.001	<0.001	0.0099			
NDAb	<0.001	<0.001	0.0018	0.0039	0.0005	0.0039	0.0053	0.0039	<0.001	0.0005		
NDPbAb	<0.001	<0.001	<0.001	<0.001	<0.001	<0.001	<0.001	<0.001	<0.001	<0.001	0.0007	

S3 – SOD (day 7)

P values derived from Mann Whitney U test for day 7 of the experiment												
	NT	D	DPb	N	Ab	NAb	ND	NDPb	DAb	DPbAb	NDAb	NDPbAb
NT												
D	0.0028											
DPb	0.0106	0.0770										
N	<0.001	0.7304	<0.001									
Ab	0.0244	<0.001	<0.001	<0.001								
NAb	0.4363	0.0315	0.7304	0.0040	0.2581							
ND	<0.001	<0.001	<0.001	<0.001	<0.001	<0.001						
NDPb	<0.001	0.2581	<0.001	<0.001	<0.001	<0.001	<0.001					
DAb	<0.001	0.6665	<0.001	0.0315	<0.001	<0.001	<0.001	0.7962				
DPbAb	<0.001	0.8633	<0.001	0.4363	<0.001	0.0008	<0.001	0.0188	0.0400			
NDAb	<0.001	0.7132	<0.001	0.7787	<0.001	<0.001	<0.001	<0.001	0.0729	0.9821		
NDPbAb	<0.001	0.3401	<0.001	0.2973	<0.001	0.0078	<0.001	<0.001	0.0056	0.1135	0.0591	

S4 – SOD (day 14)

P values derived from Mann Whitney U test for day 14 of the experiment												
	NT	D	DPb	N	Ab	NAb	ND	NDPb	DAb	DPbAb	NDAb	NDPbAb
NT												
D	<0.001											
DPb	0.1615	<0.001										
N	<0.001	>0.9999	<0.001									
Ab	0.0012	<0.001	0.0244	<0.001								
Nab	0.7304	<0.001	0.7304	<0.001	<0.001							
ND	<0.001	<0.001	<0.001	<0.001	<0.001	<0.001						
NDPb	<0.001	0.0400	<0.001	0.0400	<0.001	<0.001	0.0008					
D-Ab	>0.9999	<0.001	0.2581	<0.001	0.0005	0.5457	<0.001	<0.001				
DPbAb	<0.001	0.0078	0.0005	0.0056	<0.001	<0.001	<0.001	<0.001	<0.001			
NDAb	<0.001	0.1903	<0.001	0.1615	<0.001	<0.001	<0.001	0.0333	<0.001	0.1135		
NDPbAb	<0.001	<0.001	<0.001	0.0012	<0.001	<0.001	<0.001	<0.001	<0.001	0.8906	0.4894	

S5 – GST (day 7)

P values derived from Mann Whitney U test for day 7 of the experiment												
	NT	D	DPb	N	Ab	NAb	ND	NDPb	DAb	DPbAb	NDAb	NDPbAb
NT												
D	<0.001											
DPb	<0.001	<0.001										
N	<0.001	0.2581	<0.001									
Ab	<0.001	<0.001	<0.001	<0.001								
NAb	<0.001	<0.001	0.3333	<0.001	0.3333							
ND	<0.001	<0.001	<0.001	<0.001	<0.001	<0.001						
NDPb	<0.001	<0.001	<0.001	<0.001	<0.001	<0.001	<0.001					
DAb	<0.001	<0.001	<0.001	<0.001	<0.001	0.0939	0.0039	<0.001				
DPbAb	<0.001	0.0503	<0.001	0.0939	<0.001	<0.001	<0.001	<0.001	<0.001			
NDAb	<0.001	<0.001	<0.001	0.0106	<0.001	<0.001	<0.001	<0.001	<0.001	0.0503		
NDPbAb	<0.001	<0.001	0.0019	<0.001	<0.001	<0.001	<0.001	<0.001	<0.001	<0.001	<0.001	

S6 - GST (day 14)

P values derived from Mann Whitney U test for day 14 of the experiment												
	NT	D	DPb	N	Ab	NAb	ND	NDPb	DAb	DPbAb	NDAb	NDPbAb
NT												
D	<0.001											
DPb	<0.001	<0.001										
N	<0.001	<0.001	0.0244									
Ab	<0.001	<0.001	<0.001	<0.001								
NAb	<0.001	0.0503	<0.001	<0.001	<0.001							
ND	<0.001	<0.001	<0.001	0.0007	<0.001	<0.001						
NDPb	<0.001	<0.001	<0.001	<0.001	<0.001	<0.001	<0.001					
DAb	0.8433	<0.001	<0.001	<0.001	<0.001	<0.001	<0.001	<0.001				
DPbAb	<0.001	<0.001	<0.001	<0.001	<0.001	<0.001	<0.001	<0.001	<0.001			
NDAb	<0.001	0.1135	<0.001	<0.001	<0.001	0.3865	<0.001	<0.001	<0.001	<0.001		
NDPbAb	<0.001	<0.001	0.1615	0.0005	<0.001	<0.001	<0.001	<0.001	<0.001	<0.001	<0.001	

S7 - MDA (day 7)

P values derived from Mann Whitney U test for day 7 of the experiment												
	NT	D	DPb	N	Ab	NAb	ND	NDPb	DAb	DPbAb	NDAb	NDPbAb
NT												
D	<0.001											
DPb	<0.001	0.0005										
N	<0.001	<0.001	<0.001									
Ab	<0.001	<0.001	<0.001	<0.001								
NAb	<0.001	0.0770	0.5457	<0.001	<0.001							
ND	<0.001	<0.001	<0.001	<0.001	<0.001	<0.001						
NDPb	<0.001	<0.001	<0.001	0.0315	<0.001	<0.001	<0.001					
DAb	<0.001	<0.001	<0.001	0.8633	<0.001	<0.001	<0.001	0.0106				
DPbAb	<0.001	<0.001	<0.001	0.0770	<0.001	<0.001	<0.001	0.0008	0.0056			
NDAb	<0.001	<0.001	<0.001	<0.001	<0.001	<0.001	0.0040	0.0019	<0.001	<0.001		
NDPbAb	<0.001	<0.001	<0.001	<0.001	<0.001	<0.001	0.6210	<0.001	<0.001	<0.001	0.0028	

S8 – MDA (day 14)

P values derived from Mann Whitney U test for day 14 of the experiment												
	NT	D	DPb	N	Ab	NAb	ND	NDPb	DAb	DPbAb	NDAb	NDPbAb
NT												
D	<0.001											
DPb	<0.001	0.0315										
N	<0.001	<0.001	<0.001									
Ab	0.0142	<0.001	<0.001	<0.001								
NAb	<0.001	0.0142	0.0002	<0.001	<0.001							
ND	<0.001	<0.001	<0.001	<0.001	<0.001	<0.001						
NDPb	<0.001	<0.001	<0.001	<0.001	<0.001	<0.001	<0.001					
DAb	<0.001	<0.001	<0.001	<0.001	<0.001	<0.001	<0.001	0.3401				
DPbAb	<0.001	0.0244	<0.001	<0.001	<0.001	0.7962	<0.001	<0.001	<0.001			
NDAb	<0.001	<0.001	<0.001	0.0003	<0.001	<0.001	0.7962	<0.001	<0.001	<0.001		
NDPbAb	<0.001	<0.001	<0.001	0.7962	<0.001	<0.001	<0.001	0.0012	0.0056	<0.001	0.0005	
